# Assessment of catatonia and inter-rater reliability of three instruments: a descriptive study

**DOI:** 10.1186/s13033-021-00505-8

**Published:** 2021-11-22

**Authors:** Zukiswa Zingela, Louise Stroud, Johan Cronje, Max Fink, Stephan van Wyk

**Affiliations:** 1 Executive Dean’s Office, Faculty of Health Sciences, Nelson Mandela University, Postnet Suite 274, Private Bag X13130, Humewood, Port Elizabeth, 6013 South Africa; 2 Department of Psychology, Nelson Mandela University, Port Elizabeth, South Africa; 3 Department of Psychiatry, Stony Brook University, New York, USA; 4 Department of Psychiatry, Walter Sisulu University, Mthatha, South Africa

**Keywords:** Catatonia, Assessment, Screening Tool, Bush Francis Catatonia Rating Scale

## Abstract

**Background:**

Clinical assessment of catatonia includes the use of diagnostic systems, such as the Diagnostic and Statistical Manual, Fifth Edition (DSM-5) and the International Classification of Disease, Tenth Revision (ICD-10), or screening tools such as the Bush Francis Catatonia Screening Instrument (BFCSI)/Bush Francis Catatonia Rating Scale (BFCRS) and the Braunig Catatonia Rating Scale. In this study, we describe the inter-rater reliability (IRR), utilizing the BFCSI, BFCRS, and DSM-5 to screen for catatonia.

**Methods:**

Data from 10 participants recruited as part of a larger prevalence study (of 135 participants) were used to determine the IRR by five assessors after they were trained in the application of the 14-item BFCSI, 23-item BFCRS, and DSM-5 to assess catatonia in new admissions. Krippendorff’s α was used to compute the IRR, and Spearman’s correlation was used to determine the concordance between screening tools. The study site was a 35-bed acute mental health unit in Dora Nginza Hospital, Nelson Mandela Bay Metro. Participants were mostly involuntary admissions under the Mental Health Care Act of 2002 and between the ages of 13 and 65 years.

**Results:**

Of the 135 participants, 16 (11.9%) had catatonia. The majority (92 [68.1%]) were between 16 and 35 years old, with 126 (93.3%) of them being Black and 89 (66.4%) being male. The BFCRS (complete 23-item scale) had the greatest level of inter-rater agreement with α = 0.798, while the DSM-5 had the lowest level of inter-rater agreement with α = 0.565. The highest correlation coefficients were observed between the BFCRS and the BFCSI.

**Conclusion:**

The prevalence rate of catatonia was 11.9%, with the BFCSI and BFCRS showing the highest pick-up rate and a high IRR with high correlation coefficients, while the DSM-5 had deficiencies in screening for catatonia with low IRR and the lowest correlation with the other two tools.

## Background

Catatonia shows a wide range of prevalence in different populations, from less than 10% to just above 60% [1–3]. This may be influenced by factors that include the assessment tools used to screen for catatonia and the inter-rater reliability (IRR) among clinicians undertaking the assessment. In this descriptive study, we determined the IRR among a group of five mental health professionals who assessed participants for catatonia in an inpatient acute mental health unit. This is the first study to assess catatonia in South Africa. This is in the background of very limited studies on catatonia in South Africa. At the time of this study, there were only three papers published on catatonia in South Africa [4]. The results of our study are therefore likely to add to the limited knowledge on the presentation of catatonia and its assessment. Due to the dearth of studies on catatonia in South Africa, our study could potentially have a wide-ranging application for future research on prevalence studies for catatonia and could also be a source of comparison for future studies and findings. It may also provide useful guidance for clinicians in the assessment of catatonia.

### Assessment of catatonia

Tools or diagnostic systems, such as the Diagnostic and Statistical Manual, Fifth Edition (DSM-5) or the International Classification of Diseases, Tenth Revision (ICD-10), help guide the clinical examination when assessing catatonia [5, 6]. The validity and IRR of assessment tools are important considerations that may influence the pick-up rate of catatonia, which makes it important to choose a valid assessment tool with acceptable IRR [7, 8]. The Bush Francis Catatonia Screening Instrument (BFCSI)/Bush Francis Catatonia Rating Scale (BRCRS) meets these criteria as an acceptable screening tool because it has shown good IRR and has been successfully used at study sites to screen new admissions for catatonia [4, 7, 8]. Several studies have also indicated that the BFCSI is a reliable and valid screening tool for catatonia [1–4, 7–9].

#### Relevance of catatonia in clinical practice

Catatonia can be caused by various factors, including severe mental disorders, neurological and medical conditions, and substances [1–11]. In addition to this complexity, there have been challenges in the recognition, diagnosis, and treatment of catatonia in recent years [12]. Due to the possibility of adverse outcomes associated with catatonia, which may at times be fatal, clinicians need to be skilled at assessing catatonia despite these complexities and irrespective of the cause. This ensures that it is recognized early and is not missed and would make it possible for treatment to be initiated early to avoid protracted catatonia, which can potentially lead to a worse outcome [2, 13, 14]. All these factors imply that clinicians need to be skilled in the assessment and diagnosis of catatonia in order to treat patients promptly and effectively.

#### Relevance of catatonia in the current diagnostic systems

Catatonia was not classified as a separate diagnosis in diagnostic systems such as the DSM and ICD, but as a specifier for other mental conditions. This has continued in the latest versions of these diagnostic systems and may have contributed to the lack of clinical focus on catatonia and possible lack of recognition by clinicians in the past. In both the DSM-5 and ICD-10, catatonia is reflected as a specifier for mood disorders and schizophrenia [2, 4, 7, 10–17]. It is also reflected as being linked to another medical condition or as a condition with unspecified etiology under ‘catatonia not otherwise specified’ [4]. In ICD-11, catatonia is now classified as a subchapter, where in ICD-10, it was classified as lower in the hierarchy [18–20]. The impact and significance of this change in clinical practice remains unclear. The anticipated impact is that the changes in the diagnostic and classification systems could possibly improve their utility for clinicians in the assessment of catatonia.

#### The importance of diagnosing catatonia across all levels of mental healthcare

Although the lack of recognition of catatonia in the clinical setting remains a challenge, concerted efforts have been made to increase catatonia recognition, diagnosis, and treatment [2, 9]. The remaining challenges in recognition of catatonia could be due to several factors, including the assumption that catatonia is rare, the fluctuation and periodic nature of the presentation in some patients with waxing and waning of symptoms during an episode, and the misinterpretation of catatonic symptoms as being “put on” by patients to gain attention [2, 8, 9].

Rapid intervention to achieve resolution of catatonia is crucial to ensure that it has a good response and is resolved successfully [1–3]. This may help prevent the progression to chronic or more severe catatonia with potentially life-threatening complications. A longer duration of catatonia has been associated with a worse response to treatment [1–3]. Timely recognition and treatment may help to avoid the more serious complications of catatonia, some of which are potentially fatal. These include autonomic instability, bed sores, contractures, aspiration pneumonia, malnutrition, dehydration, renal failure, deep vein thrombosis, and pulmonary embolus [2, 14–16].

Due to the various causes of catatonia, it often presents in inpatient psychiatric settings; however, it is also seen in accident and emergency settings and medical and neurological settings. When catatonia is missed, diagnosis is delayed and has serious implications for treatment response. This is because a good response to treatment has been observed in patients with acute onset [1–3]. It is therefore crucial to sensitize staff in these settings to be alert to the possibility of catatonia and to know how to assess patients accurately, using reliable tools that are sensitive and relatively easy to apply for the average clinician working in such settings. This study examined the IRR of three screening tools in an inpatient acute mental health setting and provides insight into the applicability of these tools when used by nursing and medical staff who work in this setting [21].

#### Relevance of this research for mental health systems and policy

The current mental health policy framework and strategic plan of South Africa (2012–2020) is up for review and does not consider catatonia as an entity that requires focus when considering mental disorders across all levels of care [17]. The relevance of catatonia in the next mental health policy framework and for future planning of mental health systems is that one needs to consider the needs associated with this condition and ensure that electroconvulsive therapy (ECT) is factored into the next policy as a required resource, since it is an effective treatment for catatonia. Thus, ensuring that the availability of ECT is written into such a policy would have a bearing on access to this intervention for patients presenting with catatonia in the future.

### Aim of the study

We aimed to determine the IRR in the assessment of catatonia by five trained assessors, using the DSM-5 and BFCSI/BFCRS to screen for catatonia.

## Methods

### Study design

This was a prospective, descriptive study that utilized a quantitative method. Data from 135 participants were collected from September 2020 to February 2021 to screen for catatonia, with the first 10 participants suspected of having catatonia each screened by five assessors to determine the IRR and concordance rates of the assessment tools.

### Setting

The setting was a 35-bed acute mental health unit in Dora Nginza Hospital, Nelson Mandela Bay Metro, which is a city with a population of 1.2 million people in the Eastern Province of South Africa [22]. The city has high unemployment and morbidity, as well as mental illness rates [23]. Mental health services at the mental health unit (MHU) include 24-h care for persons who present with acute mental illness requiring inpatient treatment and ECT for those who may need it. The MHU receives referrals from other departments within the hospital, as well as local clinics and district hospitals. The unit is serviced by a multidisciplinary team that includes a psychiatrist, medical doctors training to become psychiatrists (registrars/residents), professional nurses, two clinical psychologists, two social workers, and an occupational therapist. There are no specialized radiological services such as computed tomography scans, nor is there a neurology department within the hospital, which bears relevance to the subject of our research in that all patients requiring these additional services would need to be referred to other local hospitals that offer these services. Lastly, patients who may require a longer period of admission beyond the first 2 to 4 weeks are usually referred to a local psychiatric hospital.

### Outline of the study process

The research team screened all new admissions for catatonia. Assessors were two psychiatry residents, both with 2 years of experience in psychiatry, and three mental health professional nurses each with a background of more than 10 years working in mental health services. Assessors were informed of new admissions on the day of admission, except for those participants admitted over the weekend. Assessments, including the application of the BFCRS, took place on the same day for admissions that occurred before 1200 h midday or, the day after for later admissions. For weekend or public holiday admissions, the assessment was undertaken on the first working day thereafter. The BFCRS was therefore either applied on the same day of admission in most cases or a day or two later.

### Participants

A total of 148 patients were admitted to the MHU during the first 6-months of the study, and 135 (91.2%) of them agreed to participate. Most were involuntary admissions under the Mental Health Care Act of 2002 [24]. The patients were between the ages of 13 and 65 years, due to the lack of child, adolescent, and geriatric inpatient-specific services in the region. The first 10 of the 135 participants who were suspected to have catatonia, as identified by the junior doctor-on-call, were recruited for the in-depth IRR assessment using the BFCSI/BFCRS and DSM-5 for evaluation by the five assessors.

### Measures and assessment tools

#### Comparing the BFCSI and DSM-5 in the assessment of catatonia

The BFCSI was developed by Bush et al. as a 14-item screening tool for catatonia. To assess the severity of catatonia, its use is complemented by completion of the full 23-item BFCRS if two or more signs of catatonia are present [5, 7]. The BFCRS has also been recommended by Sienaert et al. for routine use because of its good reliability, validity, and relative ease of application [9].

In a study by Sarkar et al. on the assessment of catatonia and IRR using four different instruments, more cases with catatonia were identified when applying the full BFCRS scale compared to the DSM-5, and IRR was demonstrated to be good (α = 0.779) [4, 10–12, 25]. Like this study, the number of assessors in our study was five, consisting of three professional nurses and two psychiatry residents.

### Training of assessors

The principal investigator trained the five assessors on how to use the BFCSI/BFCRS and DSM-5 to assess catatonia. The training consisted of explaining the meaning of the terms used in the BFCSI/BFCRS and DSM-5 to describe signs of catatonia, and practical demonstrations of how to elicit and document the DSM-5 diagnostic criteria for catatonia and the 14- and 23-items in the BFCSI/BFCRS. Training also included how to uniformly capture the data on the data form and supervised practice sessions on each other and on practice participants under the guidance of the principal investigator.

#### Quality assurance of the assessment process

Quality assurance of the assessment process was undertaken through observed practice sessions. Assessors practiced on each other during the training, under observation and guidance of the principal investigator, who is a psychiatrist skilled in the assessment of catatonia. Furthermore, assessors conducted the application of the BFCRS during the first week under continued observation by the principal investigator to ensure that the application was performed correctly.

An IRR with a Krippendorff’s α in the range of 0.61 and 0.8 (during the 1 week’s scoring) in at least one of the screening tools was considered acceptable for the assessors to continue with the scoring for the rest of the study participants in the broader prevalence study [11]. Each of the 10 participants was assessed by all assessors on the same day for the IRR part of the study, or on days as close as possible to each other, to minimize differences due to fluctuation of symptoms. The definitions for catatonia that were inherent to the screening tools were accepted as the cut-off points for the diagnosis of catatonia, that is, the presence of two or more symptoms in the BFCSI/BFCRS and presence of three or more symptoms in the DSM-5. In cases where the assessors identified possible missed catatonia, the treating doctor was provided with any additional information found during the participant assessments to allow for a review of the patient’s clinical case and management.

### Sampling

Convenience sampling of all patients admitted to the MHU over the first 6-month period of the study was undertaken. Inclusion criteria were all participants who were admitted as inpatients to the MHU and consented to participate in the study. All patients who refused consent were excluded.

The number of patients that were expected to be admitted during the first 6 months of the study period was approximately 130, based on unit admission figures over the previous 6 months. This was due to the restrictive effects of the coronavirus disease 2019 (COVID-19) outbreak on admission rates and bed occupancy in the MHU. The restrictions were mainly because the new admissions required screening for COVID-19, and the patients had to be admitted in separate spaces within the unit that would allow for social physical distancing. This resulted in a reduced bed capacity compared to the usual 35-bed occupancy and lower admission rates. Six months was chosen as the approximate duration it would take to recruit the 10 patients for the IRR evaluation, given the effect of COVID-19 on admission rates. The part of the study that focused on determining the IRR was limited to 10 participants suspected of having catatonia. This is because this aspect of the study necessitated that each of the five assessors had to assess each of the 10 participants. This meant that even though there were 10 participants assessed for determination of the IRR, the total number of assessments performed was 50. The limit of 10 participants was thus set owing to financial implications and limitations related to study funding.

### Data management and analysis

To determine the IRR, Krippendorff’s α was computed for each screening tool. Krippendorff’s α allows multiple assessors to assess the same set of patients [11]. To make use of this method, each tool used to assess each patient for catatonia was recoded into a binary variable reflecting either the presence or absence of catatonia. A higher Krippendorff’s α reflects a higher rate of agreement between assessors. The presence of catatonia observed per tool was compared using Spearman’s correlation coefficient (ρ) [15]. Quantitative data on the assessment and presentation of catatonia, as well as demographic and clinical data such as age, sex, ethnicity, diagnosis, and substance use, were summarized using descriptive statistics, with categorical variables presented using frequency and contingency tables. Descriptive statistics were calculated for the samples used to assess IRR and the rest of the samples for the prevalence study.

## Results

### Overall findings

In total, there were 148 admissions during the first 6 months of the study, and 135 (91.2%) of them consented to be screened for catatonia. Of the 135 participants, 16 screened positive for catatonia over 6 months (September 2020 to February 2021), which yielded a prevalence rate of 11.9%. Of the 10 participants assessed for the IRR part of the study, six (60%) had catatonia according to the BFCRS.

### Clinical and demographic findings

The demographics of the total sample of 135 participants are shown in Table 1. The majority of participants [92 (68.1%)] were between 16 and 35 years old, with 126 (93.3%) of them being Black and 89 (66.4%) male. The remaining demographic information is presented in Table 1.

**Table 1:** Demographic profile of participants

Variables	N (%)
Age	
16–35 years	92 (68.1%)
36–65 years	41 (30.4%)
> 65 years	2 (1.5%)
Sex	
Female	44(32.8%)
Male	89 (66.4%)
Unknown	1 (0.8%)
Ethnicity	
Black	126 (93.3%)
Colored	7 (5.2%)
White	1 (0.75%)
Unknown	1 (0.75%)

The most common psychiatric diagnosis in the whole sample was bipolar disorder, which was diagnosed in 49 (36.3%) participants, followed closely by schizophrenia with 41 (30.4%) participants, and substance-induced psychotic disorder with 20 (14.8%) participants. The rest was made up of substance-induced bipolar disorder, major depressive disorder, or unknown diagnoses. On analysis of the data for the 16 participants with catatonia, there were three statistically significant associations observed at both the 10% and 5% levels. These associations were between BFCSI and diagnosis (Statistic = 10.268, p = 0.067), BFCSI and previous catatonic diagnosis (Statistic = 8.072, p = 0.015), and finally, BFCRS and previous catatonic diagnosis (Statistic = 7.328, p = 0.022). The association between BFCSI and diagnosis revealed that of the 15 participants assessed to have catatonia via this tool, eight were diagnosed with schizophrenia.

### Interrater reliability findings

Of the 135 participants, the first 10 thought to have catatonia based on the initial assessment by the admitting doctor were assessed by all five assessors to determine the IRR. This means that there were 50 assessments in total that were performed by the five assessors, which made up the bulk of the data used for analysis to determine the IRR.

The yield from the 50 assessments conducted on the 10 participants was that six (60%) participants were found to have catatonia based on the BFCSI/BFCRS and DSM-5 criteria. Eight (80%) of the 10 participants were aged 35 years or younger, and eight (80%) were male.

The resulting Krippendorff’s α values are listed in Table 2. BFCRS and BFCSI had higher Krippendorff’s α values compared to the DSM-5. The BFCSI (14-items scale) and BFCRS (complete 23-item scale) had the greatest level of inter-rater agreement, each with an α = 0.798, while the DSM-5 had the lowest level of inter-rater agreement with α = 0.565. The correlation coefficients reflecting the presence of catatonia in each diagnostic method are shown in Table 3. The highest correlation coefficients were observed between the BFCRS and BFCSI.

**Table 2:** Krippendorff’s alpha for the rating scales

Screening Tool	Krippendorff's alpha
BFCRS	0.798
DSM-5	0.565
BFCSI	0.798
Current Catatonia	0.778

**Table 3:** Correlations among the rating scales

	BFCRS Complete	DSM-5	BFCRS Screen
BFCRS Complete	1	0.564	1.000
DSM-5		1	0.564
BFCRS Screen			1

## Discussion

The assessment tools with the best IRR, as reflected by a Krippendorff’s α of 0.798 and a correlation coefficient of 1, were the BFCSI and BFCRS. The DSM-5 had the lowest Krippendorff’s α at 0.565 and a correlation coefficient of 0.564 with both BFCRS and BFCSI. Several studies have indicated the validity, ease of application, and good IRR of the BFCSI/BFCRS as an assessment tool for catatonia in clinical settings [1–5, 7, 8]. None of the studies had evaluated the use of these screening tools in an African setting. Similarly, this study also reflects that BFCRS is the most sensitive instrument for picking up catatonia, even in a South African acute mental health unit setting. The DSM-5, on the other hand, had a much lower IRR score and correlated poorly with the other screening tools, indicating shortcomings in identifying catatonia. This implies that, specifically for the assessment of catatonia, it may not be as useful in the clinical setting [1–4].

Thus, the findings of this study support the utility of the BFCSI/BFCRS as an assessment tool in an acute mental health setting and highlight the importance of using such tools over and above the DSM-5 when assessing patients with catatonia. Another important finding of our study is that it was possible to upskill mental health nurses to assess new admissions for catatonia after only one training session with a specialist psychiatrist, achieving a good level of IRR with a validated tool. Bearing in mind that in clinical settings, psychiatrists and medical doctors have expertise in the diagnosis and treatment of severe and enduring mental illness, the usefulness of our study findings is that it indicates a potential screening role for appropriately trained nursing staff in primary care. The study findings may also have potential implications for resource-limited inpatient settings, such as the study site, which provide treatment for serious mental illness where catatonia might present. Since catatonia may also be present in accident and emergency, internal medicine, and neurology departments, the implications of these findings may potentially be applicable in clinical environments beyond MHUs. This is especially important because catatonia may also be present in delirious patients more likely to be seen in these clinical settings [16].

### Implications of findings for mental health systems

This study demonstrates that professional nurses working in mental health can be upskilled with one session to apply easily available and free screening tools such as the BFCSI/BFCRS, to assess patients with catatonia. Given the potential for progression to more severe catatonia, which may be fatal if untreated, our finding suggests there is a prime opportunity to review training and guidelines for screening at the primary care level. The results of our study represent the first step towards gathering evidence on the assessment of catatonia in South Africa, which in turn may be available to policy makers when they weigh allocation of resources for mental health interventions. Moreover, assessment of catatonia using the BFCSI did not add much time to the clinical assessment, and assessors still managed to maintain a high IRR with good correlation with the BFCRS, making BFCSI a potentially useful tool for screening for catatonia in similar inpatient settings. In other words, our findings suggest that use of the BFCSI/BFCRS to screen for catatonia may improve the overall capacity and ability to assess for catatonia, thus diminishing the reliance on the clinician’s clinical acumen alone, which may help to avoid missing cases of catatonia in such settings.

### Forgotten clinical syndromes and rediscovering catatonia

As much as prevalence studies on catatonia have revealed that catatonia is not a rare clinical syndrome, the defocus of the current diagnostic and classification systems on catatonia as an entity of clinical significance and importance has landed catatonia amid the forgotten syndromes in psychiatry [26, 27]. This has left clinicians in a difficult position where modern training in psychiatry no longer focuses on equipping clinicians with skills to identify and categorize these presentations and may therefore be a contributor to the apparent assumption that a condition like catatonia is rare in modern times, despite the evidence to the contrary regarding the prevalence rates of catatonia [1–3, 6, 7, 9, 11, 13].

In the themed issue of the *International Review of Psychiatry*, Fiorillo and Ventriglio stated that new forms of psychiatric disturbances have come about, which are linked to modernized culture and social challenges, and have replaced many psychiatric disorders that were previously long-established. Examples of these forgotten syndromes include de Clarambault syndrome, Capra’s syndrome, and Fregoli syndrome to name a few [26, 27]. Furthermore, Onofa et al., in their study on the reliability and clinical utility of ICD-11 in Nigeria, recognized the importance of classification and diagnostic systems, acknowledging that the value of such classification systems is dependent on their utility and acceptability to those who may need to apply them in the clinical setting [19]. Taking all of this into consideration, the findings of our study could contribute to the assessment of catatonia in a clinical setting. This is especially so in resource-limited settings where the first point of contact at a clinic or other health institution may often be a nurse. By arming nursing personnel with validated tools to screen for catatonia and training them in the application of such tools, it would theoretically be possible for them to identify which patients need further assessment by a skilled doctor to diagnose catatonia or other underlying conditions.

## Study limitations

The limitations of this study include the small sample size, the number of assessment tools for catatonia, which did not include the Braunig Catatonia Rating Scale or the ICD-10, and the fact that the analysis only focused on the presence or absence of catatonia on admission and did not include cases where catatonia might have developed during the rest of the inpatient period.

## Conclusion

The findings of this study support findings from other studies on catatonia, which indicate that catatonia is not a rare condition, with a prevalence of 11.9%. The findings of this study also show that the BFCRS and BFSCI are reliable screening tools for catatonia with good IRR, and that the DSM-5 has notable deficits which limit its utility in the assessment of catatonia in the clinical setting [4, 5].

## Data Availability

All data are stored at the study site and are available on request from the lead author at the Walter University, Department of Psychiatry and Human Behavioral Sciences.

## Abbreviations

BFCRS: Bush Francis Catatonia Rating Scale
BFCSI: Bush Francis Screening Instrument
DSM-5: Diagnostic and Statistical Manual Fifth Edition
ICD-10: International Classification of Diseases Tenth Revision
IRR: Inter-rater reliability
